# Neuroprotective Potential of Secondary Metabolites from *Melicope lunu-ankenda* (Rutaceae)

**DOI:** 10.3390/molecules24173109

**Published:** 2019-08-27

**Authors:** Zeinab Abdulwanis Mohamed, Enas Mohamed Eliaser, Emanuela Mazzon, Patrick Rollin, Gwendoline Cheng Lian Ee, Ahmad Faizal Abdull Razis

**Affiliations:** 1 Laboratory of Molecular Biomedicine, Institute of Bioscience, Universiti Putra Malaysia, 43400 UPM Serdang, Selangor, Malaysia; 2 Department of Biology, Faculty of Science, El-Mergib University, El Khums, Libya; 3 IRCCS Centro Neurolesi “Bonino-Pulejo”, Via Provinciale Palermo, Contrada Casazza, 98124 Messina, Italy; 4 Université d’Orléans et CNRS, ICOA, UMR 7311, BP 6759, F-45067 Orléans, France; 5 Chemistry Department, Faculty of Science, Universiti Putra Malaysia, 43400 UPM Serdang, Selangor, Malaysia; 6 Laboratory of Food Safety and Food Integrity, Institute of Tropical Agriculture and Food Security, Universiti Putra Malaysia, 43400 UPM Serdang, Selangor, Malaysia; 7 Department of Food Science, Faculty of Food Science and Technology, Universiti Putra Malaysia, 43400 UPM Serdang, Selangor, Malaysia

**Keywords:** polyphenols, coumarins, alkaloids, *Melicope lunu-ankenda*, neuroprotection, neurodegenerative diseases

## Abstract

Plant natural compounds have great potential as alternative medicines for preventing and treating diseases. *Melicope lunu-ankenda* is one *Melicope* species (family *Rutaceae*), which is widely used in traditional medicine, consumed as a salad and a food seasoning. Consumption of different parts of this plant has been reported to exert different biological activities such as antioxidant and anti-inflammatory qualities, resulting in a protective effect against several health disorders including neurodegenerative diseases. Various secondary metabolites such as phenolic acid derivatives, flavonoids, coumarins and alkaloids, isolated from the *M. lunu-ankenda* plant, were demonstrated to have neuroprotective activities and also exert many other beneficial biological effects. A number of studies have revealed different neuroprotective mechanisms for these secondary metabolites. This review summarizes the most significant and recent studies for neuroprotective activity of *M. lunu-ankenda* major secondary metabolites in neurodegenerative diseases.

## 1. Introduction

**Figure 1:**
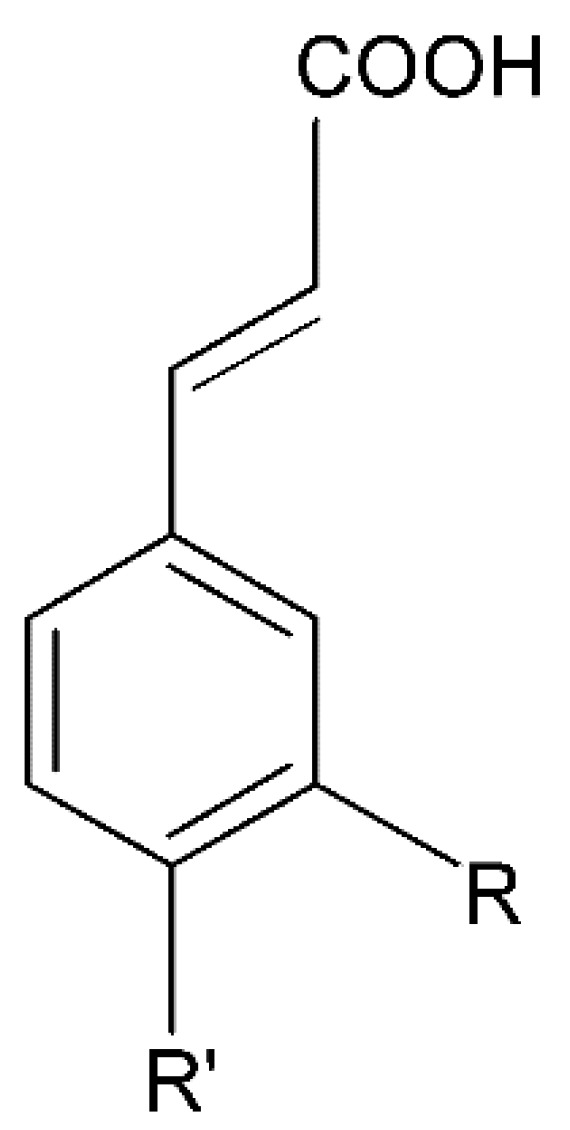
General chemical structure of hydroxycinnamic acid. Adapted from Soobrattee et al. [8].

**Figure 2:**
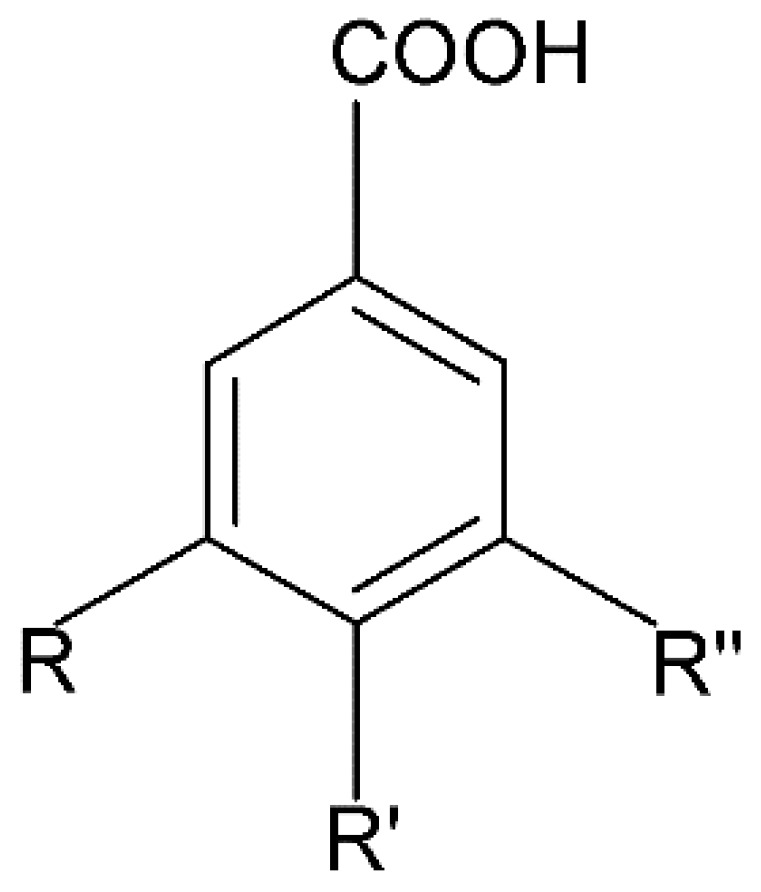
General chemical structure of hydroxybenzoic acid. Adapted from Soobrattee et al. [8].

**Figure 3:**
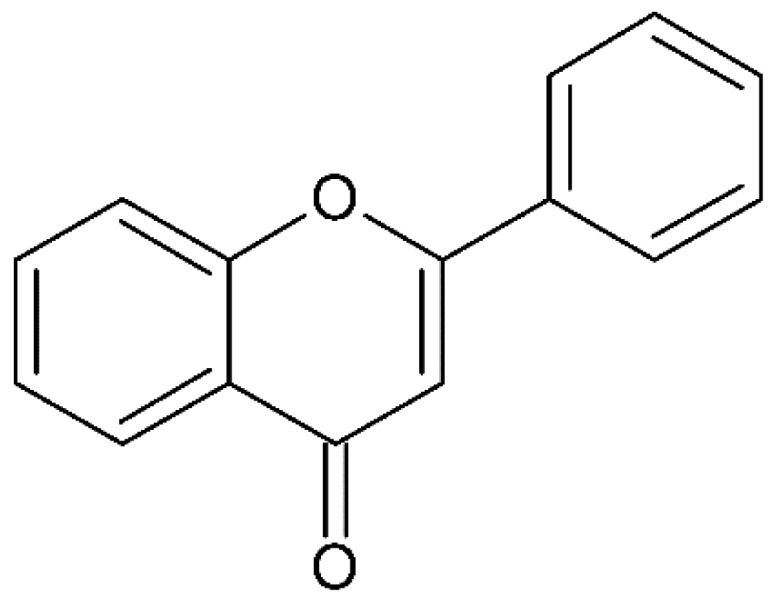
General chemical structure of flavonoids. Adapted from Soobrattee et al. [8].

Millions of people worldwide are currently affected by neurodegenerative diseases (NDDs) the most common being Alzheimer’s disease (AD). NDDs are more common in countries with a high average life expectancy, and this has drawn the attention of researchers due to their social and economic impacts [1]. NDDs, such as Alzheimer’s disease (AD), Parkinson’s disease (PD) and Huntington’s disease (HD), are characterized by progressive loss of function and structure of the nerve cells [2]. Although the actual cause of NDDs is still unknown, there are some common factors contributing to NDDs which include neuro-inflammation, β-amyloid (Aβ) aggregation, neurofibrillary tangle formation, oxidative stress, and impairment of mitochondrial function [2]. However, ageing is considered as the greatest risk factor for NDDs [2]. Considering the fact that NDDs represent chronic and incurable conditions, bioactive compounds isolated from medicinal plants have become the best option to prevent and alleviate neurological disorders. Phytochemicals have become interesting therapeutic candidates due to their various biological properties including, but not limited to anti-oxidant, anti-inflammatory activities, neuroprotective effects and chemical characteristics such as direct uptake of free radicals, and modulation of enzymes associated with oxidative stress [1]. *Melicope lunu-ankenda* (*Evodia lunu-ankenda* (Gaertn.) Merr) also known locally in Malaysia as “tenggek burung” is a species of the genus *Melicope*, which belongs to the Rutaceae family. It is commonly distributed in tropical Asia, Malaysia, Indonesia, Sri Lanka, Philippines, Thailand and Australia. *M. lunu-ankenda* is widely used as a medicinal plant with its leaves usually consumed as food seasoning and salad. It has also been used to treat hypertension, diabetes mellitus, fever, menstrual disorders, and as a tonic [3,4]. Previous studies have isolated anti-inflammatory, anti-oxidative and immunomodulatory compounds from *M. lunu-ankenda* leaves [5]. Different studies on *M*. *lunu-ankenda* leaves have led to the isolation of interesting secondary metabolites such as polyphenols including cinnamic acid derivatives (Figure 1), benzoic acid derivatives (Figure 2) and flavonoids (Figure 3), coumarins and alkaloids [3]. Those metabolites extracted and isolated from plants have shown neuroprotective activity such as polyphenols from yerba mate (*Ilex paraguariensis*) and green tea (*Camellia sinensis*), respectively reported to exhibit memory enhancing effect on dementia and antioxidant activity or to stimulate the expression of cell-survival genes respectively [6]. Coumarins from the fruit of *Psoralea corylifolia* have shown alleviation of scopolamine-induced amnesia in rats [7], whereas alkaloids from *Corydalis ternata* exhibit anti-cholinesterase and anti-amnesic activity [6]. However, not many scientific studies have been carried out on secondary metabolites isolated from the *M. lunu-ankenda* plant to evaluate its neuroprotective activity. More investigations are required in order to further explore the molecular diversity of bioactive compounds in *M. lunu-ankenda* and their biological activities.

## 2. Polyphenols 

**Figure 4:**
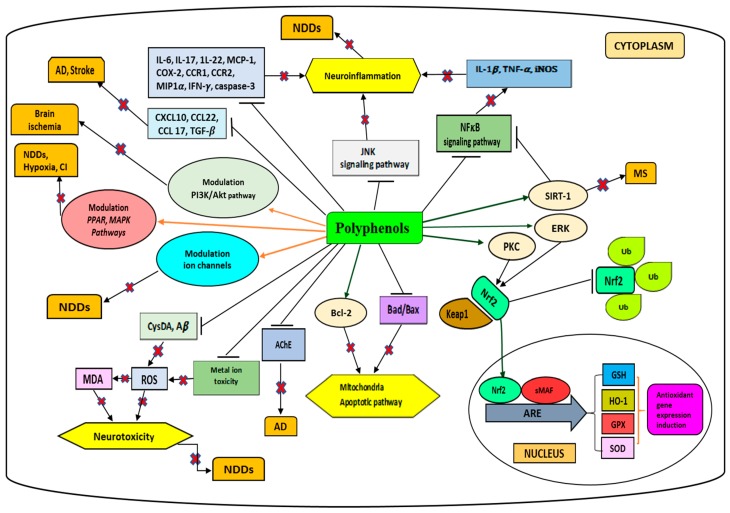
The mechanisms of neuroprotective activity exhibited by polyphenols. ARE, antioxidant response element; GSH, glutathione; ROS, reactive oxygen species; Nrf2, nuclear factor (erythroid-derived 2)-like 2; NF-κB, nuclear factor kappa-light-chain-enhancer of activated B cells; iNOS, inducible nitric oxide synthase; HO-1, heme oxygenase 1; Sesn2, sestrin 2; GCL, glutamate-cysteine ligase; GSTs, glutathione S-transferase; Keap1, Kelch-like ECH-associated protein 1; sMAF, proto-oncogene response element; SOD, superoxide dismutase; IL, interleukin; IFN-γ, interferon-gamma; GPx, glutathione peroxidase; TNF-α, tumor necrosis factor-alpha; TGF-β, transforming growth factors β; COX-2, cyclooxygenase-2; MCP-1, monocyte chemoattractant protein-1; SIRT-1, silent mating type information regulation 2 homolog 1; JNK, c-Jun N-terminal kinase; Bcl-2, B-cell lymphoma-2; Bad, BCL2-associated agonist of cell death; BAX, BCL2-associated X protein; Aβ, amyloid beta; AChE: acetylcholinesterase; ERK, extracellular signal-regulated kinase; PKC, protein kinase C; PI3K, phosphatidylinositol 3-kinase; Akt, protein kinase B; PPAR, peroxisome proliferator-activated receptor; CXCL10, chemokine (C-X-C motif) ligand 10; CCL, chemokine (C-C motif) ligand; CCR, chemokine receptor; MIP1α, macrophage inflammatory protein 1 α; MAPKs, mitogen-activated protein kinases; CI, cerebral ischemia; cysDA, cysteinyldopamine; MDA, malondialdehyde; Ub, Ubiquitin.

Phenolic compounds are widely distributed secondary metabolites in plants. They are ubiquitous in plant food such as vegetables, cereals, fruits, nuts and beverages including tea and cocoa [8]. *M. lunu-ankenda* has shown high total phenolic content (TPC) [9]. Natural phenolic compounds can range from simple compounds such as phenolic acids and flavonoids to complex biomolecules such as lignins and tannins [8]. Natural antioxidants, particularly phenolics, have potential therapeutic effects against neurological disorders, inflammatory diseases and also ageing [8]. The therapeutic effects of phenolic compounds are mostly attributed to their antioxidant activity, scavenging of free radicals, metal ion chelation, gene expression modulation and interaction with signaling pathways of the cell [8]. The anti-radical and antioxidant activities of phenolic compounds are dependent upon the number and position of hydroxyl groups attached to the aromatic ring [10]. Several studies conducted experimentally and epidemiologically have revealed that polyphenols modulate anti-oxidant and anti-inflammatory signaling pathways [11]. Thus, they have ability to attenuate two neurological disorder hallmarks including inflammation and oxidative stress [11]. The common neuroprotective mechanisms of polyphenols are summarized in Figure 4.

Autophagy dysfunction represents one of the common neurodegenerative disease features characterized by accumulation of toxic protein aggregates. Polyphenolic compounds including curcumin have been demonstrated to enhance autophagy through nuclear translocation of TFEB (transcription factor EB) and inhibiting the phosphoinositide 3-kinase-AKT-MTOR signaling pathway [12]. Despite the fact that pre-clinical and clinical evidences have demonstrated the safety and beneficial effect of polyphenols compounds [13], their low bioavailability and inefficient delivery system represents a critical issue for their introduction into functional food and clinical practice [14]. Most (90%–95%) of the total polyphenol intake accumulates in the large intestinal lumen, where it is subjected to the effects of the gut microbial community enzymes, thus undergoing structural changes [15]. The bioavailability of polyphenols is dependent upon first-pass metabolism modifications, intestinal tract permeation and cell-membrane permeability and solubility. Exploring a new delivery system becomes necessary to reach the therapeutic levels of polyphenols in target organs through the bloodstream [13]. Recently, nanoparticle-based delivery systems including polymeric nanoparticles that encapsulate polyphenolic molecules as nanostructures such as nanocapsules (NCs), solid lipid nanoparticles (SLNs), nanospheres (NSs), micelles (MCs), liposomes (LSs) and cyclodextrins (CDs) are promising solution for enhancing polyphenol bioavailability and improving their therapeutic potential [14]. 

Polyphenols in *Melicope lunu-ankenda*, including phenolic acid derivatives and flavonoids (Figure 3), are described below. 

### 2.1. Phenolic Acids

Phenolic acids are the most abundant polyphenols in plants, including cinnamic acid derivatives (caffeic acid, caffeoylquinic acid, coumaric acid, ferulic acid and sinapic acid) (Figure 1) and benzoic acid derivatives (gallic acid) (Figure 2).

#### 2.1.1. Cinnamic Acid Derivatives 

Cinnamic acid derivatives are isolated from *M. lunu-ankenda* leaves. It was observed that cinnamic acid derivatives have anti-oxidant effects [16]. A study conducted on several phenolic compounds to evaluate their antioxidant capacity has shown that hydroxycinnamic acids exhibited a greater antioxidant activity than hydroxybenzoic acids [8]. Hydroxycinnamic acid derivatives showed the greatest antioxidant capacity due to the presence of the vinylogous CH=CH−COOH group (Figure 1) instead of the COOH group in benzoic acid derivatives [16].

A recent study reported the properties of new cinnamic acid-based derivatives as multifunctional cholinesterase inhibitors for Alzheimer’s disease, focused on the combination with benzyl-pyridinium salts used as scaffold to develop new AChE (acetylcholinesterase) and BuChE (butyrylcholinesterase) inhibitors [17]. The capacity of the cinnamic acid-based derivatives to inhibit AChE and BuChE activities was assessed by the spectrophotometric Ellman’s method. Cinnamic acid derivatives (IC_50_ = 12.1 nM) showed the most potent inhibitory activity against AChE than that of donepezil (IC_50_ = 40.2 nM), and exhibited the highest inhibitory activity against BuChE (IC_50_ = 1.9 μM) than that of donepezil (IC_50_ = 4.5 μM). The study also showed that the cinnamic acid-based derivatives inhibit self-induced Aβ (1–42) aggregation, and could chelate metal ions [17].

##### Caffeic Acid 

Caffeic acid [3-(3,4-dihydroxyphenyl)-2-propenoic acid] is a hydroxycinnamic acid found commonly in plant-derived foods such as coffee, vegetables and fruits. Caffeic acid possess significant biological activities including, but not limited to antioxidant and anti-inflammatory activities [18]. A study conducted on Erigeron annuus leaves demonstrated that caffeic acid was the active compound isolated from the butanol fraction of a methanolic extract, with antioxidant activity and protective effect on pheochromocytoma (PC12) cells against hydrogen peroxide-induced neurotoxicity at concentration 40 μg/mL [19]. It was also suggested that caffeic acid, as potent antioxidant and neuroprotective compound, can be used to treat neurodegenerative diseases such as AD [19].

Other studies also reported the neuroprotective activity of caffeic acid against amyloid-β (Aβ)-induced cell toxicity. Caffeic acid at 10 and 20 μg/mL has shown neuroprotective effect against Aβ-induced toxicity in PC12 cells through decreased intracellular calcium level, oxidative stress, inhibition of tau phosphorylation and phosphorylation of GSK-3β (glycogen synthase kinase-3β) [18] (Table 1). A study conducted on primary mouse cortical neurons to evaluate the neuroprotective activity of caffeic acid revealed neuroprotective effects against 5-*S*-cysteinyl-dopamine-induced damage at concentration 1 μM. It was suggested that caffeic acid could be a significant candidate for treatment of PD [20], as shown in Figure 4, and Table 1.

Alkyl esters of caffeic acid were synthesized to improve the lipophilicity of their parent compound. Their activity as antioxidant, neuroprotective capacity in PC12 cells against H_2_O_2_-induced cell injury were evaluated. Caffeic acid esters showed higher lipophilicity and antioxidant activity than their parent compound and they also exerted PC12 cells protection at 5 µM and 25 µM [21]. This study suggested that both antioxidant activity and lipophilicity of hydroxycinnamic acids are essential for inducing neuroprotective effects.

Increasing scientific evidence supports a pivotal role for HO-1 (the inducible isoform of heme oxygenase) in the resolution of acute inflammatory states, providing efficient cytoprotection against oxidative stress. Low concentrations of caffeic acid phenethyl ester showed antioxidant and anti-inflammatory properties, significantly increased HO-1 expression and heme oxygenase activity in Type 1 astrocytes (DI TNC1) at 6- or 24-hour post-treatment. Expression levels of glutathione (GSH) and glutathione disulfide (GSSG) were measured to evaluate the effect of the polyphenolic compound on the redox state of the cell. It was shown that exposure to 15 and 30 µM caffeic acid for 6 hours resulted in a significant increase in both intracellular GSH and GSSG [22]. 

An animal model of AD was used to investigate whether caffeic acid could prevent Aβ-related cognitive impairments in mice, modulate the Nrf2/HO-1 signalling pathway, and attenuate the oxidative stress in the hippocampus. C57BL/6 mice were injected with unilateral stereotaxic intracerebroventricular (i.c.v) injection of A_β1-42_O and treated one hour after brain lesion by intraperitoneal (i.p) administration of 10 mg/kg of caffeic acid showing its antioxidant, antiapoptotic and anti-inflammatory activity. The reported data denoted an increased expression of Nrf2 and HO-1, probably modulated by GSK3β activity [23], which brings significant improvement of cognitive deficits accompanied by decreasing levels of reactive oxygen species (ROS) and neuronal death in brain tissue.

The neuroprotective effect of caffeic acid was also tested in an animal model of rotenone-induced Parkinson’s disease. Caffeic acid (10 mg/kg) was able to improve dopamine level and reduce the expression of inflammatory cytokines TNF-α and IL-1β. The downregulated expression of cyclooxygenase-2 (COX-2), inducible nitric oxide synthase (iNOS) and activated B cells (NFκB) were also reported. After treatment, significant improvement in behavioural tests was observed [24].

##### Caffeoylquinic Acid

Caffeoylquinic acid (CQA) and its derivatives are phenolic natural compounds which possess neuroprotective, antioxidant effects. Recently, CQA derivatives have been reported to show neuroprotective activity in Aβ-induced PC12 cell toxicity. Moreover, CQA has a neuroprotective effect in vitro against cell death and *in vivo* against ischemia-induced cell injury. The authors suggested that CQA derivatives need more investigation to understand the mechanism through which they exert the neuroprotective activity [25] (Figure 1). The neuroprotective effect of 3,5-*di*-O-caffeoylquinic acid (3,5-di-O-CQA) on a Aβ_1–42_ treated SH-SY5Y human neuroblastoma cell line was studied using 3-(4,5-dimethylthiazol-2-yl)-2,5-diphenyltetrazolium bromide (MTT) assays. It was shown that 3,5-di-O-CQA at 20μM exhibited neuroprotective effect on SH-SY5Y cells treated with Aβ_1–42_. This suggested that 3,5-di-O-CQA exerts a neuroprotective effect via activation of ATP production and induction of PGK1 (phosphoglycerate kinase-1) expression [25] (Table 1).

The antioxidant effects of caffeoylquinic acid were also tested against oxidative stress induced by hydrogen peroxide in SH-SY5Y neuroblastoma cells. Pre-treatment with 20 μM caffeoylquinic acid significantly increased superoxide dismutase (SOD) activities and decreased reactive oxygen species (ROS) and malondialdehyde (MDA) levels. The pre-treatment also showed anti-apoptotic effects decreasing the Bax/Bcl-2 ratio, and inhibiting caspase-3 and caspase-9 activation. Moreover, inhibition of ERK1/2 phosphorylation and increase of AKT/GSK-3β phosphorylation was also proven [26].

Chen et al. [27] have investigated the neuroprotective effects of caffeoylquinic acid derivatives in an in vivo model of cerebral ischemic injury. The results confirmed that caffeoylquinic acid inhibits apoptosis and reduces neuronal cell damage. The protective effect is related to upregulation of Bcl-2 protein and decreasing expression of Bax and NFκB1.

##### Coumaric Acid

5-*S*-Cysteinyl-dopamine (CysDA) conjugates have been demonstrated to possess neurotoxic activity. Raised levels of such neurotoxins were found in the substantia nigra of PD patients. They may attenuate dopamine uptake. A study was conducted on mouse cortical neurons to investigate the cytoprotective effect of polyphenolic compounds, including *p*-coumaric acid (CA), against CysDA neurotoxicity [20]. CA at 1 µM exhibited significant neuroprotective activity against CysDA-induced injury in vitro. Moreover, the cytoprotective effect induced by CA was found to be higher than for (+)-catechin and (−)-epicatechin [20] (Figure 4). The neuroprotective activity of CA on spinal cord ischemia/reperfusion injury (SCIR) in rats was investigated. The malondialdehyde (MDA) level was demonstrated to be increased after SCIR injury. Treatment with single-dose 100 mg/kg (i.p.) CA showed spinal cord protection against lipid peroxidation by decreasing the level of MDA. Superoxide dismutase (SOD) activity increased due to development of oxidative stress after ischemia/reperfusion. The effect of SOD can be attenuated if oxidative stress persists. The SCIR model treated with CA showed increase in SOD activity, which may be due to the protection effect of CA against ROS [28] (Figure 4, Table 1).

Mitochondrial biogenesis is activated by neurons as a response to mitochondrial damage, ROS induction, and ATP impairment in spinal cord ischemia (SCI). CA protects the mitochondrial function by upregulating mitochondria biogenesis. The level of nuclear respiratory factor-1 (NFR1) decreases if the oxidative stress persists for a long time. Interestingly, treatment with CA induced an increase of NFR1 level. Mitochondrial biogenesis induction by CA could play a key role for mitochondrial function improvement in NDDs. This study demonstrated the neuroprotective effect of CA on SCIR in rat [28].

The neuroprotective effects of CA were tested in an in vitro model of Alzheimer disease induced with Aβ in PC12 (rat adrenal medulla) cells. The previous treatment of cells with CA appreciably inhibited ROS generation in a dose-dependent manner ranged from 0.5 μM up to 125 μM with consequent inhibitory effect on caspase-3 [29].

##### Ferulic Acid

An in vitro study was conducted by Kanski et al. [30] *in vitro* to evaluate the antioxidant capacity of ferulic acid (FA) against hydroxyl- and peroxyl radical-induced cell injury in a synaptosome model and hippocampal neuronal cell culture. It was shown that FA at 50 µM greatly attenuates free radical injury in neuronal cell culture and reduces lipid peroxidation and ROS level in the synaptosomal system. The effects of FA were compared with those of vanillic acid, coumaric acid and cinnamic acid. FA showed greater antioxidant and free radical scavenging activities than the three other phenolic acids. Moreover, bioavailability studies have shown that FA exhibits the convenient pharmacokinetic characteristics [30] (Table 1). A strong reduction of neuroinflammation was observed as a result of FA administration in the transgenic APPswe/presenilin 1 (PS1)dE9 (APP/PS1) AD mouse model. This study found that FA-treatment at concentration 5.3 mg/kg/day for 6 months significantly attenuated the frontal cortex level of Aβ deposition and interleukin-1β (IL-1β) [31] (Figure 4, Table 1).

Similar findings were disclosed through a study conducted *in vivo* on transgenic (Tg2576) AD mice model to investigate the attenuation activity of phenolic compounds including FA on Aβ aggregation [32] (Table 1). This study revealed that long-term administration of FA prevented Aβ-induced learning and memory deficits. FA exerted cytoprotective effects via upregulating heme oxygenase 1 (HO-1), SOD, extracellular signal-regulated kinase 1/2 (ERK1/2) and protein kinase B (Akt) (Figure 1), contrary to its downregulating caspases, COX-2, and iNOS [32] (Figure 4). A study conducted in vitro by fluorescence spectroscopy examination with thioflavin T and electron microscopy analysis revealed that FA dose-dependently of the order of 1–10 µM inhibited formation and extension of β-amyloid fibrils (fAβs). Moreover, it showed destabilization of preformed fAβs [33].

A study conducted on 21-month-old rats supplemented with sodium ferulate (SF; 100–200 mg/kg for 4 weeks) has shown inhibition of interleukin-1β(IL-1β) and upregulation of ERK1/2 and Akt [31] (Figure 4). Moreover, SF has the ability to attenuate caspase activation induced by Aβ. SF pre-treatment attenuated the activation of caspase-3, caspase-7, caspase-9 cascade in rats injected with Aβ [32]. FA administrated subcutaneously at 5 mg/kg concentration for 6 days was reported to decrease brain damage from oxidative injury induced by glutathione deprivation [31].

##### Sinapic Acid 

Sinapic acid (3,5-dimethoxy-4-hydroxycinnamic acid; SA) is one of the most distributed hydroxycinnamic acids in plants. Studies have shown that SA has various biological activities such as antioxidant, anti-inflammatory and anti-anxiety. The antioxidant and radical scavenging activities of SA were evaluated using the 2,2-diphenyl-1-picrylhydrazyl (DPPH**˙**) test. It was shown that SA could inhibit 33.2% of the DPPH at a concentration of 20 μM, which is close to the caffeic acid scavenging activity (49.6%). In a similar study conducted at 0.5 SA/DPPH molar ratio, 88.4% inhibition was found for SA, which was comparable to caffeic acid with a 92.7% scavenging activity. The authors claimed that SA represents an attractive candidate as potent antioxidant agent [34].

The neuroprotective activity of SA was investigated in Aβ_1–42_ protein-induced AD mouse model. Terminal deoxynucleotidyl transferase-mediated dUTP nick-end labeling (TUNEL) assay and Nissl staining were used: Nissl-positive cells were increased and TUNEL-positive cells were decreased through SA treatment in the hippocampal CA1 region. After injection of Aβ_1–42_ protein in the hippocampal CA1 region, caspase-3 activation was analyzed, revealing an increase of the number of caspase-3-positive cells. SA treatment 10 mg/kg/day (p.o.) significantly decreased the number of caspase-3-positive cells. It was shown that SA attenuated Aβ_1–42_ protein-induced cell death in the hippocampal (CA1) region, inhibited microglia and astrocyte activation and reduced expression of iNOS, memory impairment. The neuroprotective effect of SA was exerted via anti-inflammatory, antioxidant and attenuation of apoptosis [35] (Table 1). Those results suggested that SA could be a suitable agent for the treatment of AD. 

The neuroprotective effect of SA in the 6-hydroxydopamine (6-OHDA)-induced PD rat model was evaluated by Zare et al. [36]. This study showed that, at a dose of 20 mg/kg, SA was able to prevent loss of substantia nigra pars compacta (SNC) dopaminergic neurons, reduce MDA level and inhibit iron reactivity (Figure 4). The authors claimed that the neuroprotective activity of SA was exerted via inhibition of oxidative stress and level of nigral iron [36] (Table 1).

#### 2.1.2. Benzoic Acid Derivatives

##### Gallic Acid

Gallic acid (3,4,5-trihydroxybenzoic acid; GA) is the most potent antioxidant and antiradical among the benzoic acid derivatives. The striking antioxidant, antiradical activities observed with gallic acid are linked to the presence of three hydroxyl groups bonded to the aromatic ring [9].

The neuroprotective activity of oral GA against 6-OHDA-induced oxidative stress at doses of 50 mg/kg, 100 mg/kg and 200 mg/kg for 10 days in PD rat model was examined by Mansouri et al. [37]. This study showed GA to exhibit neuroprotective activity due to its antioxidant power. Moreover, after GA treatment, an increase in the passive avoidance memory, in glutathione peroxidase (GPx) level and a decrease in the level of MDA were observed [37] (Figure 4, Table 1). The neuroprotective activities of GA derivatives are not only dependent on their antioxidant capacity but depend more on their molecular polarities as shown by Lu et al. [38] in a study conducted on gallic acid derivatives to investigate their structure, antioxidant capacity, neuroprotective effect relationship using liposome system and human SH-SY5Y cell line. This study revealed that the compounds with proper hydrophobicity and high antioxidant capacity are the best option to treat NDDs due to their effectiveness to prevent the oxidative stress injury.

The anti-inflammatory and neuroprotective properties of GA were tested in a rat model of traumatic brain injury (TBI). The inflammatory pathway induced by TBI conditions determined the expression of pro-inflammatory cytokines such as interleukin-1β (IL-1β), interleukin-6 (IL-6) and tumor necrosis factor-α (TNF-α). These factors induce neuronal death and blood–brain barrier disruption accompanied by severe clinical disorders and cognitive impairment. It was shown that pre-treatment with GA reduced IL-1β, IL-6 and TNF-α in cellular brain extract 48 hours after TBI [39].

Another study also reported the neuroprotective effects of GA against in vitro model of neurotoxicity induced by 100 μM glutamate in primary rat cortex neurons (RCN) [40]. Significant results were obtained using the dose of 50 μg/mL of GA. The levels of *N*-acetyl aspartate (NAA), a marker of neuronal integrity, has been monitored. A significant elevation in NAA level was observed in GA-treated cortex neurons and the glutamate-induced neuronal damages could be prevented. GA improved antioxidant enzyme activity, showing reduced levels of nitric oxide concentration and estimating a significantly improved level of glutathione and SOD activity [40]. GA-treated RCN cells showed attenuation of the inflammatory cascades reporting the inhibition of TNF-α and reducing IL-β and IL-6 expression. GA treatment also resulted in upregulation of IGF-1 with consequent inhibition of NF-κB activation [40].

### 2.2. Flavonoids

Neuroinflammation is characterized by microglia activation, which induces pro-inflammatory cytokine level and causes neuronal disorders. Flavonoids are among the most important polyphenolic compounds that have been demonstrated to exhibit neuroprotective activity. This has been ascribed to their ability to modulate NFκB and mitogen-activated protein kinases (MAPKs) signaling pathways and attenuate the expression of COX-2, IL-6, IL-1β and TNF-α (Figure 4). Authors recently suggested that natural flavonoids require more investigation to evaluate and improve their bioavailability [41].

#### 2.2.1. Quercetin

Quercetin is one of the most studied flavonoids namely because of its antioxidant activity. Quercetin neuroprotective activity against induced oxidative cell damage in rat cortical cell cultures and its antioxidant effects were evaluated. A study shows that quercetin with IC_50_ 10.67 µg/mL attenuated xanthine/xanthine oxidase (X/XO) or H_2_O_2_-induced neuronal cell damage. It was demonstrated that quercetin is a potent antioxidant and has neuroprotective effects in rat cortical cell cultures [42]. Quercetin showed neuroprotective activity in PC12 cells at 50 and 100 μM by attenuating nitric oxide (NO) production and iNOS expression against 6-OHDA-induced cell death. This study also demonstrated neuroprotection effect of quercetin in zebrafish against 6-OHDA-stimulated dopaminergic neuron loss, by downregulating pro-inflammatory gene expression including IL-1β, COX-2 and tumor necrosis factor-alpha (TNF-α) [43] (Figure 4, Table 1).

An in vivo study was conducted on mice treated with quercetin (5, 10 mg/kg daily) for 8 weeks using step through and Morris water maze tests. These mice showed memory and learning ability improvement. Quercetin was also found to increase SOD and decrease MDA [44] (Table 1). It has been demonstrated to modulate IL-1β, interleukin 6 (IL-6) and TNF-α, to activate silent mating type information regulation 2 homolog1 (SIRT1), to attenuate Aβ_1-40_ and Aβ_1-42_ by downregulation of nuclear factor kappa-light-chain-enhancer of the NFκB pathway (Figure 1) and block sodium channels. Quercetin was also found to exhibit a neuroprotective effect by attenuating metalloproteinases (MMP), and induce glutathione peroxidase (GPx), Na^+^/K^+^-ATPase. Cognitive ability improvement and neuronal cell protection against trimethyltin-induced neuronal cell injury by attenuated acetylcholinesterase (AChE) was observed for quercetin. Quercetin has shown ability to down regulate c-Jun, interferon-γ inducible protein and c-Jun N-terminal kinase (JNK) [11] (Figure 4). It has also been reported to stimulate the nuclear factor erythroid 2-factor 2 (Nrf2) in cerebellar granule cells exposed to oxidative stress [44], thus leading to raise neuronal level of glutathione that has the ability to restore redox homeostasis (Figure 4)**.**

The protective action of quercetin was investigated by Jembrek et al. [45] in an in vitro model of oxidative stress induced by H_2_O_2_ in P19 neurons. The study reported that quercetin at 150 μM favoured neuronal survival through prevention of neuronal death, by increase of Bcl-21/2 signalling pathways and modulation of Akt and ERK. The neuroprotective effect of quercetin was also accompanied by suppression of p53 up regulation and Bcl-2 down regulation [45].

#### 2.2.2. Isorhamnetin

According to Al-Zuaidy et al. [3], isorhamnetin was one of the main bioactive compounds identified in the ethanolic extract from *M. lunu-ankenda*. In vivo and in vitro studies demonstrated the antioxidant and anti-inflammatory activities of isorhamnetin. In a study conducted on experimental stroke-induced mice to investigate the neuroprotective effects of this flavonoid, it was found that isorhamnetin at 5 mg/kg improved the blood brain barrier (BBB) function, upregulated Nrf2/HO-1, downregulated NR1, reduced oxidative stress, inflammation, cerebral edema, NO production and suppressed iNOS (Figure 4). This study also revealed that isorhamnetin protected the brain against ischemic injury in mice [46] (Table 1).

In addition, a study conducted by Ishola et al. [47] evaluated the effects of isorhamnetin in an animal model of amnesia induced by scopolamine. Pre-treatment with isorhamnetin (1, 5 or 50 mg/kg, p.o.) resulted in satisfactory behavioural tests, such as recognition and orientation. A molecular evaluation of hippocampus and prefrontal cortex cellular extraction showed that mice pre-treated with isorhamnetin presented a significant decrease in acetylcholinesterase activity and significant elevation of brain-derived neurotrophic factor (BDNF) level. The study disclosed that isorhamnetin reduced the generation of malondialdehyde and nitrite. Moreover, an increase of glutathione (GSH), superoxide dismutase (SOD) and catalase (CAT) activities in the prefrontal cortex and in the hippocampus was observed [41].

#### 2.2.3. Catechin

Polyphenolic catechins are known to exhibit neuroprotective activity. Epicatechins inhibited neuronal injury induced by oxidized low-density lipoprotein in mouse-derived striatal neurons [42]. Besides, it has been shown that neuronal protection against oxidative damage and stroke occurred through modulation of Nrf2/ARE/HO1 [11] (Figure 4). Catechins exerted neuroprotection to mesencephalic neurons against 6-OHDA-induced neurotoxicity. (−)-Epigallocatechin-3-gallate (EGCG) showed neuroprotective activity against 1-methyl-4-phenylpyridinium- and 6-OHDA-induced neurotoxicity in human neuroblastoma cells [48].

As reported in a recent study, an in vitro model using primary rat hippocampal neurons tested the neuroprotective properties of EECG after exposure to corticosterone (CORT) [49]. The authors reported that the molecular mechanisms underlying CORT-induced neuronal lesions were dependent on the inhibition of ERK1/2 and PI3K/AKT pathways. CORT is involved in decreasing the expression of PGC-1α, a transcription coactivator that stimulates mitochondrial biogenesis, with a reduction of ATP production. The results obtained demonstrated that EGCG at concentrations between 0.1 and 5 μmol/L exerts neuroprotection of corticosterone-induced damage in hippocampal neurons by restoring ERK1/2 and PI3K/AKT signaling pathways and promoting PGC-1α expressions and ATP production in neurons primary hippocampal [49].

Neuroprotective effects of catechin hydrate against oxidative stress and ischemic injury in rat brain was demonstrated by attenuated expression of NFκB, IL-1β and TNF-α (Table 1). EGCG was found to protect neuron cells by inhibiting matrix metalloproteinases (MMP) in cerebral ischemia mouse model. ECGC ability to attenuate oxidative stress, monocyte chemotactic protein (MCP-1), IL-6, IL-1β, chemokine C-C motif ligand (CCL22, CCL17, CXCL10) and JNK was also reported. Moreover, it also showed upregulation of SOD and glutathione peroxidase (GP_X1_) and effected iron chelation in SH-SY5Y cells along with attenuation of caspase-3, BCL2-associated agonist of cell death (Bad) and BCL2-associated X protein (Bax) [11] (Figure 4). Catechins have been demonstrated to upregulate various signaling pathways that protect neuronal cells against oxidative injury and inflammation. They also showed stimulation of PKC and apoptosis prevention [6] (Figure 4). 

## 3. Coumarins

**Figure 5:**
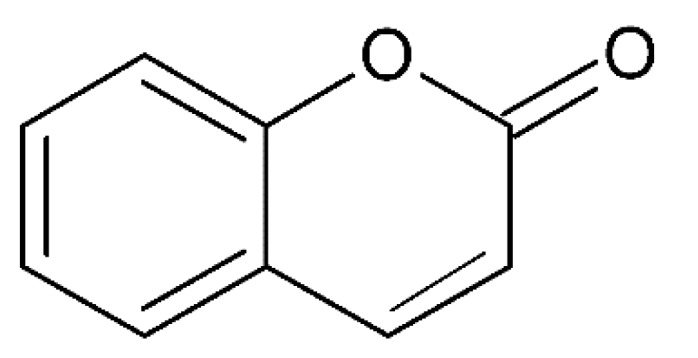
General chemical structure of coumarins. Adapted from Jameel et al. [50].

Coumarins (2*H*-1-benzopyran-2-ones) (Figure 5) are important phytochemicals widely distributed in the plant kingdom which belong to the benzo-α-pyrone class [50]. They represent a large group of natural products which can be found in many plant species, mainly in the Rutaceae family [51]. They are distributed throughout the plant parts such as fruits, flowers and leaves [52]. Coumarins possess a wide range of biological activities including but not limited to anti-oxidant, anti-AD and anti-inflammatory [53], anticonvulsant and neuroprotective activities [54].

Different studies have revealed that plant-derived coumarins possess acetylcholinesterase (AChE) enzyme inhibitory activity, Aβ anti-aggregating activities as well as memory restorative effects. Such pharmacological properties are important in AD management [7]. Scopoletin, 6-deoxyhaplopinol, marmesin, melilunumarin A (7-(4-hydroxy-3-methylbutoxy) coumarin), melilunumarin B (7-(3-methoxycarbonylbutoxy) coumarin), umbelliferone, bergapten are coumarins isolated from *M. lunu-ankenda* [4]. Scopoletin, esculetin and umbelliferone are coumarins that have been demonstrated to have antioxidant activities [55].

### 3.1. Scopoletin

Scopoletin (7-hydroxy-6-methoxycoumarin) is an antioxidant compound widely found in *M. lunu-ankenda* leaves [4]. Scopoletin isolated from *Tilia amurensis* showed significant neuroprotective effects against glutamate-induced neuron cell injury in HT22 cells at concentrations ranging from 10 μM, 50 μM, and 100 μM. It was suggested that scopoletin may act as a neuroprotective agent in the treatment of AD [56] (Table 1). The antioxidant activity of scopoletin from *Sinomenium acutum* was evaluated in another study, which revealed that dose-dependent 2.5–100 μM scopoletin has the ability to scavenge the superoxide anion generated by xanthine/xanthine oxidase, without attenuation of xanthine oxidase [57]. The authors suggested that scopoletin may find use in the prevention of health disorders associated with oxidative injury.

A Huntington’s disease (HD) model was induced by 3-nitropropionic acid (3-NP) in Wistar rats. Among all secondary metabolites extracted from *Convolvulus pluricaulis* Choisy and tested, only scopoletin showed neuroprotective effects. Pre-treatment with scopoletin (20 mg/kg) induces moderate loss of body mass and has self-beneficial effects on locomotor activity. Molecular evaluation showed reduction of MDA and nitrite at cellular level. Restoration of SOD and GSH was also observed [58].

In vitro model of neurotoxicity induced by Aβ in human neuronal cell line (SH-SY5Y) and inflammatory animal model induced by 12-*O*-tetradecanoyl phorbol-13-acetate (TPA) were used to investigate the anti-Alzheimer’s potential effect of *Bouvardia ternifolia*. From the hydroalcoholic extracts (60% ethanol: 40% water), the maximum inhibition of acetylcholinesterase (38.43 ± 3.94 %) was exhibited by the scopoletin-containing fraction. Scopoletin (1.25 ± 0.37 mg) showed anti-inflammatory effects by reducing inflammation by >70% in the TPA-induced ear edema. The data also indicated percentage of inhibition of lipid peroxidation (%) by increasing concentrations of scopoletin [59].

### 3.2. Auraptene

**Figure 6:**
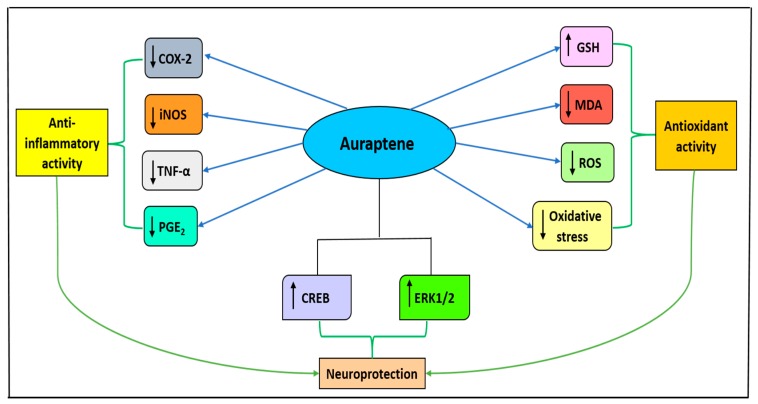
The mechanisms of neuroprotective activity exhibited by auraptene. COX-2, cyclooxygenase-2; iNOS, inducible nitric oxide synthase; TNF-α, tumor necrosis factor-alpha; GSH, glutathione; SOD, superoxide dismutase; MDA, malondialdehyde; PGE_2,_ prostaglandin E2; CREB, cAMP response element-binding protein; ERK1/2, extracellular signal-regulated kinase 1 or 2.

Auraptene (7-geranyloxycoumarin, AUR) is a widely distributed prenyloxycoumarin in the plant kingdom, particularly in species belonging to the family Rutaceae [60]. A study conducted on a transient global ischemia mouse model demonstrated the neuroprotective activity of AUR in ischemia, with results indicating its ability to protect neuronal cells by inhibition of COX-2 expression and suppression of the inflammatory response [61] (Table 1). An in vitro study using rat PC12 cells to evaluate neurotrophic effects of AUR, demonstrated that the molecule effectively activated ERK1/2, cAMP response element binding protein (CREB) and induced neurite outgrowth in PC12 cells at concentration 30 μM [62], as shown in Figure 6, Table 1.

AUR was reported to inhibit COX-2, iNOS and TNFα expression in mouse macrophage cells induced by lipopolysaccharide (LPS). It was also found to protect neuronal cells by inhibition of oxidative stress and to attenuate the prostaglandins E2 (PGE_2_) level. In contrast, a study using permanent bilateral occlusion of the common carotid arteries (2VO) rat model demonstrated that AUR 25, 8 and 4 mg/kg possess neuroprotective activity, attenuating MDA level and increasing GSH level in cortex and hippocampus [63], as shown in Figure 6.

### 3.3. Esculetin

Esculetin (6,7-dihydroxycoumarin) has been shown to exert different biological effects such as anti-inflammatory, antioxidant and anti-apoptotic activities [64]. An in vivo study conducted by Wang et al. [64] demonstrated for the first time the neuroprotective effects of esculetin on cerebral ischemia/reperfusion (I/R) injury in mouse model. It was evidenced that esculetin exerted its anti-apoptotic effects by increasing the level of anti-apoptosis protein Bcl-2 and attenuating the expression of Bax (apoptosis-related proteins) (Table 1).

## 4. Alkaloids

**Figure 7:**
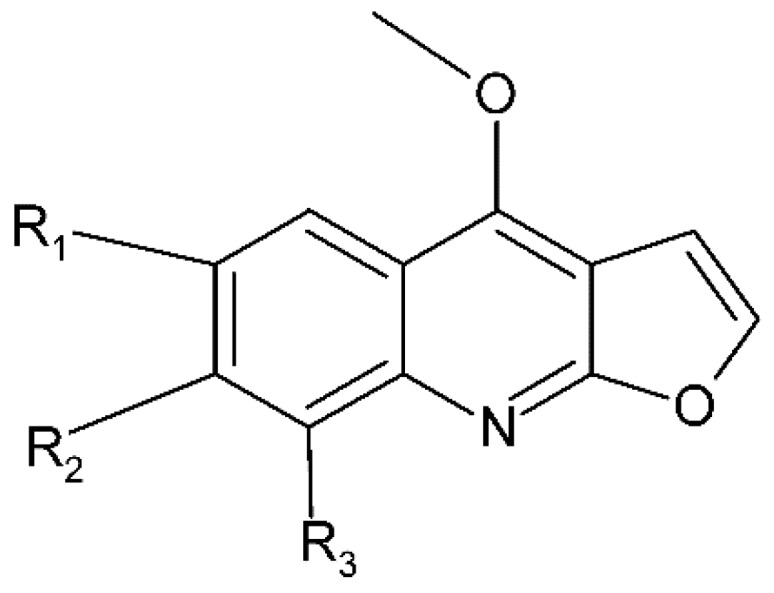
General chemical structure of furoquinoline alkaloids. Adapted from Wansi et al. [65].

Alkaloids are a class of nitrogen-containing secondary metabolites widely distributed in plants. They are found in different plant parts, particularly in the leaves [65] and they have been reported as one of the most effective neuroprotective agents [66]. The botanical family Rutaceae is particularly rich in alkaloids [61], and so is *M. lunu-ankenda*. Quinoline alkaloids (buchapine, melicarpinone, 3-(3,3-dimethylallyl)-4-(3,3-dimethylallyloxy)-2(1H)-quinolinone) and furoquinoline alkaloids (Figure 7) (roxiamines A, B, C, evolitrine, dictamnine, kokusaginine, γ-fagarine, skimmianine) have been isolated from M. lunu-ankenda. Among them, skimmianine, evolitrine, and melicarpinone were demonstrated to possess anti-inflammatory effects [4].

### 4.1. Skimmianine

Skimmianine is one of the major bioactive compounds found in the leaves of *M. lunu-ankenda* [4]. Samples of skimmianine isolated from *Ruta graveolens* L. (Rutaceae) were studied to evaluate the anti-inflammatory activity of the alkaloid in the carrageenan-induced rat paw edema model. This study indicated that skimmianine at a dose of 5 mg/kg body weight possess anti-inflammatory effects and reduces the TNF-α and IL-6 mRNA levels. It also causes a decrease of the activities of COX-2, NO and reduces PGE2 and lipid peroxidation levels (Table 1). Finally, the study also disclosed that skimmianine may play a role as a therapeutic agent in inflammatory diseases [67].

### 4.2. Evolitrin

A study was conducted by Lal et al. [68] on carrageenan-induced rat paw edema, adjuvant-induced arthritis in a rat model, to evaluate the anti-inflammatory effect of evolitrine, isolated from *M. lunu-ankenda*. At a concentration of 20 mg/kg, evolitrine inhibited 57% of carrageenan-induced edema. Moreover, at 100 and 200 mg/kg, it significantly inhibited adjuvant-induced arthritis [68] (Table 1). This study demonstrated the anti-inflammatory effect of evolitrine.

The results of a recent study revealed that two crude extracts aqueous (AELA) and 70% ethanol crude extracts (EELA) and alkaloid fraction of *Acronychia pedunculata* leaves as well as evolitrine isolated from AF-EELA possess anti- oedema activity [69]. Evolitrine was identified as the major alkaloid present in the active alkaloid fraction of EELA. The optimally effective anti-inflammatory dose of evolitrine was determined in a paw oedema animal model induced by carrageenan. Evolitrine showed the maximum percentage inhibition of oedema (78%) at the third hour for the highest tested dose of 60 mg/kg b.w [69].

## 5. Chromenes

**Figure 8:**
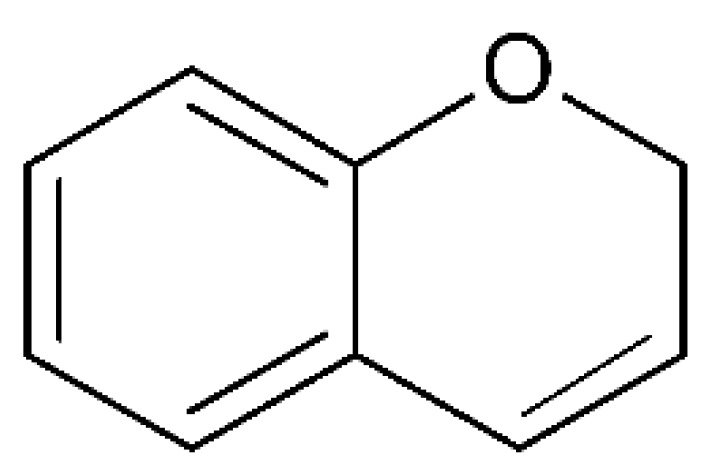
General chemical structure of chromenes. Adapted from Johnson et al. [5].

Evodione and leptonol, two major chromene-type compounds in the volatile oil and extracts of *M. lunu-ankenda* have been reported to exert different biological activities. Good antioxidant activity was demonstrated for leptonol, whereas moderate anti-inflammatory activity was found for both chromene-type compounds [5] (Table 1, Figure 8). A summary of in vitro and in vivo studies as well as the mechanisms of action of some hydroxycinnamic acids, hydroxybenzoic acids, flavonoids, coumarins, alkaloids, and chromenes as neuroprotective, anti-oxidant, and anti-inflammatory agents with their chemical structures are shown in Table 1.

## 6. Conclusions

The strategies of using phytochemicals as bio-alternative agents for NDDs therapy has become necessary as plant natural compounds show more effectiveness and fewer side effects than synthesized medicines. A large number of scientific investigations have demonstrated against NNDs the therapeutic benefits of herbal medicines, which can be ascribed to the presence of secondary metabolites such as polyphenols, coumarins and alkaloids. Those metabolites are the major phytochemicals that are distributed in different parts of *Melicope lunu-ankenda,* a plant widely used in folk medicine to treat several health disorders. Researchers have ascertained the therapeutic potential of *M. lunu-ankenda,* and its biological activities were mainly attributed to antioxidant and anti-inflammatory effects. This sheds light on the fact that *M. lunu-ankenda* may become a promising plant for prevention and treatment of neurological disorders. However, the neuroprotective activity of *M. lunu-ankenda* and mechanisms through which it exerts its activity need to be explored in more detail.

## Figures and Tables

**Table 1:** Chemical structures and mechanisms of action as neuroprotective, antioxidant, and anti-inflammatory agents of some hydroxycinnamic acids, hydroxybenzoic acids, flavonoids, coumarins, alkaloids, and chromenes found in *Melicope lunu-ankenda*.

Group	Compound	Chemical Structure	NDDs	Mechanism of Action	Ref.
Hydroxycinnamic acids					
	Caffeic aid	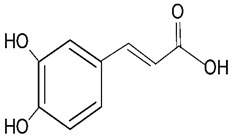	AD PD	↓oxidative stress, ↓calcium influx, ↓phosphorylation of GSK-3β (*in vitro*); PC12 cell line ↓formation of CysDA (in vitro); primary mouse cortical neurons	[18] [20]
	Ferulic acid	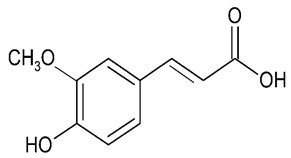	NDDs particularly AD AD AD	↓oxidative stress (in vitro); synaptosomal and neuronal cell culture ↓Aβ aggregation, ↓IL-1β (*in vivo*); APP/PS1 AD mouse model ↓ Aβ aggregation (*in vivo*); Tg2576 AD mouse model	[30] [31] [32]
	Caffeoylquinic acid (3,5-di-O-CQA)	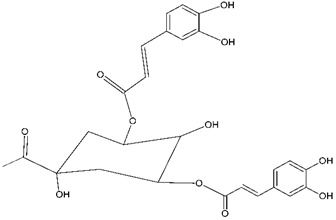	NDDs	induction of PGK1 (in vitro); SH-SY5Y cell line	[25]
	Coumaric acid	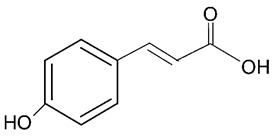	NDDs particularly SCIR	↓ MDA, ↑ SOD (in vivo); rat model	[28]
	Sinapic acid	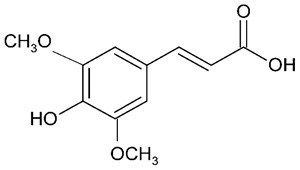	NDDs particularly AD PD	↓ Aβ1–42, ↓caspase-3, ↓apoptosis (in vivo); mouse model ↓oxidative stress, ↓MDA, ↑ SOD, ↓iron level (in vivo); rat model	[35] [36]
Hydroxybenzoic acid					
	Gallic acid	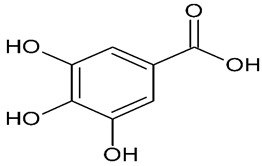	PD	↓ MDA, ↑ GPx (in vivo); rat model	[37]
Flavonoids					
	Quercetin	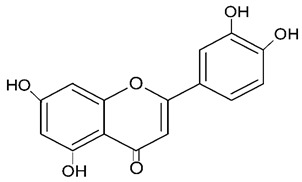	NDDs	↓NO, ↓iNOS (in vitro); PC12 cell line ↓IL-1β, ↓COX-2, ↓TNF-α (in vivo); zebrafish model ↓ MDA, ↑ SOD (in vivo); mouse model	[43] [43] [44]
	Isorhamnetin	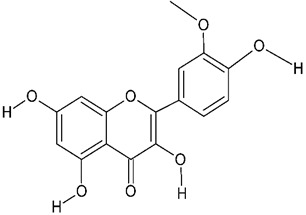	Ischemic stroke	↓NR1, ↓oxidative stress, ↑ Nrf2/HO-1 ↓ iNOS, ↓ NO (in vivo); mouse model	[46]
	Catechin	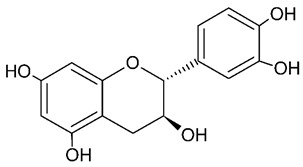	Brain ischemia	↓ NF-κB, ↓IL-1β, ↓TNF-α (in vivo); rat model	[11]
Coumarins					
	Scopoletin	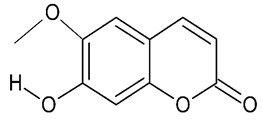	AD	antioxidant (in vitro); HT22 cell line	[56]
	Auraptene	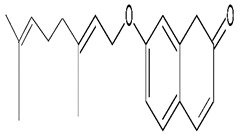	CI NDDs	↓inflammatory response, ↓COX2 (in vivo); mouse model ↑ERK1/2, ↑CREB (in vitro); PC12 cell line	[61] [61]
	Esculetin	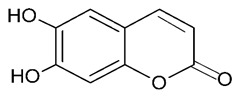	Cerebral I/R	↓ apoptotic, ↑ Bcl-2, ↓ Bax (in vivo); mouse model	[64]
Alkaloids					
	Skimmianine	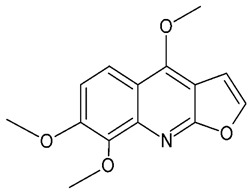	NNDs	↓ TNF-α, ↓COX2, ↓ PGE2 (in vivo); rat model	[67]
	Evolitrine	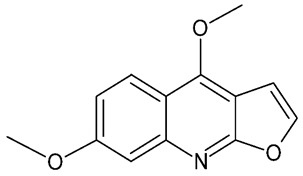	NNDs	anti-inflammatory (in vivo); rat model	[68]
Chromenes					
	Evodione	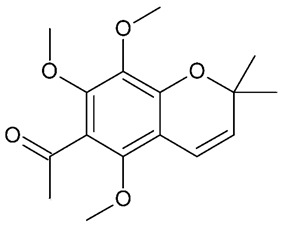	Anti- inflammatory	anti-inflammatory	[5]
	Leptonol	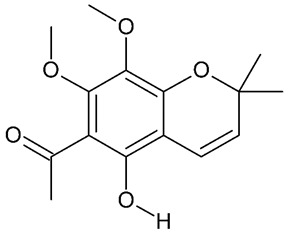	Antioxidant anti-inflammatory	Antioxidant anti-inflammatory	[5]

NDDs, neurodegenerative disease; AD, Alzheimer’s disease; PD, Parkinson’s disease; SCIR, spinal cord ischemia /reperfusion injury; CI, cerebral ischemia.
